# Knowledge of common cancers among new-entry health science students in Japan and Vietnam

**DOI:** 10.1186/s12909-023-04674-7

**Published:** 2023-10-03

**Authors:** Nhu Thi Hanh Vu, Tam Thao Tuyet Tran, Duc Trong Quach, Shunsuke Miyauchi, Mahoko Yoshida, Yuri Okamoto, Dat Minh Lu, Linh Le Tran, Mai Ngoc Luu, Toru Hiyama

**Affiliations:** 1 Department of Internal Medicine, University of Medicine and Pharmacy at Ho Chi Minh City, Ho Chi Minh, Vietnam; 2 Department of Endoscopy, Hiroshima University Hospital, Hiroshima, Japan; 3 Department of Family Medicine, University of Medicine and Pharmacy at Ho Chi Minh City, Ho Chi Minh, Vietnam; 4 Health Service Center, Hiroshima University, 1-7-1 Kagamiyama, Higashihiroshima, 739-8514 Japan

**Keywords:** Health science students, Knowledge, Common cancer

## Abstract

**Background:**

The incidence and mortality rates of cancer are rapidly increasing worldwide. This study aimed to assess the knowledge of common cancers among new-entry health science students in Japan and Vietnam, thereby providing insights for implementing appropriate medical educational interventions.

**Methods:**

This cross-sectional study was conducted among new-entry health science students at Hiroshima University, Japan, and the University of Medicine and Pharmacy at Ho Chi Minh City, Vietnam. A printed predesigned questionnaire consisting of eleven questions was distributed to the participants during the freshmen health screening at the beginning of the academic year to assess their knowledge of cancer.

**Results:**

A total of 2,748 new-entry health science students participated in the study, including 394 (14.3%) Japanese students and 2,354 (85.7%) Vietnamese students. Most cancer knowledge levels in Japanese students were significantly higher than those in Vietnamese students (p < 0.001), except for human papillomavirus (HPV) infection awareness. For this understanding, only 14.8% of Japanese students selected the correct answer, which was significantly lower than the 22.4% of Vietnamese students (p = 0.001). Both the Japanese and Vietnamese students had limited knowledge regarding the connection between HPV infection and cancer and the link between estrogen–progestogen menopausal therapy and breast cancer. Additionally, female students had better knowledge about breast, skin, and endometrial cancers than male students.

**Conclusions:**

Japanese students generally exhibited higher levels of cancer knowledge than Vietnamese students, except for HPV infection recognition. Both groups had limited knowledge regarding the connection between HPV infection and cancer and the relationship between estrogen–progestogen menopausal therapy and breast cancer.

## Introduction

Cancer remains a primary cause of death and a significant obstacle to improving life expectancy across countries worldwide [1]. According to the World Health Organization (WHO), cancer was responsible for nearly 10 million deaths in 2020 [2]. The global incidence and mortality rates of cancer are rapidly increasing, influenced by the aging and growth of the population as well as changes in the prevalence and distribution of the main cancer risk factors, several of which are associated with socioeconomic development [3].

In Japan, data from the GLOBOCAN series of the International Agency for Research on Cancer revealed 1,028,658 new cancer cases in 2020. The leading malignancies in Japan were colorectal cancer (14.4%), lung cancer (13.5%), and gastric cancer (13.5%). In contrast, Vietnam reported a much lower count of new cancer cases, totaling 182,563 in the same year. The most prevalent cancers in Vietnam were liver cancer (14.5%), lung cancer (14.4%), and breast cancer (11.8%) [4].

Enhancing public awareness and recognition of cancer is crucial for curbing the increase in cancer incidence. Developing awareness of cancer risk factors from a young age enables individuals to adopt healthier lifestyles and better regulate their health. However, several studies have indicated a lack of cancer knowledge among youth and young adults [5–7]. Insufficient awareness of cancer and its risk factors leads to delayed healthcare access and higher cancer-related mortality rates. As future physicians and healthcare providers, medical and other health science students (e.g., dental, nursing, pharmacy) constitute a significant group and play a vital role in public education. A solid understanding of cancer can empower them to raise public awareness and offer more professional suggestions for cancer prevention and early detection. Moreover, the medical curricula in Japan and Vietnam, currently constructed with a conventional approach, necessitate further scientific evidence to align educational programs more closely with student needs. Hence, evaluating the knowledge base of new-entry students could provide essential data to develop better student-centered curricula. In addition to foundational knowledge, it is necessary to highlight gaps in students’ understanding to enhance the efficacy of medical education programs.

In Japan, some studies examining cancer awareness among Japanese students revealed gaps in their understanding of the disease [8–10]. However, according to our knowledge, there is a lack of relevant research investigating the cancer knowledge of medical students in Japan and Vietnam. Hence, this study aimed to evaluate cancer knowledge among new-entry health science students in these countries. The findings might contribute to reducing cancer morbidity and mortality in both countries by providing insight into implementing more appropriate medical educational interventions.

## Methods

### Study design and participants

This cross-sectional study was conducted among new-entry health science students at Hiroshima University in Japan and the University of Medicine and Pharmacy at Ho Chi Minh City, Vietnam. Hiroshima University is a prestigious national university located in Higashihiroshima and Hiroshima, Japan. It comprises 12 undergraduate schools, including three schools in health sciences: medicine, dentistry, and pharmaceutical sciences. There were 398 health science students who entered as freshmen this year. On the other hand, the University of Medicine and Pharmacy at Ho Chi Minh City is one of the top-ranked universities of medicine and pharmacy in Vietnam. The university consists of seven faculties, including fundamental sciences, medicine, pharmacy, traditional medicine, public health, nursing and medical technology, and onto-stomatology. The total number of first-year students at this university was 2,403.

A printed predesigned questionnaire was distributed to students on the day of freshmen health screening at the beginning of the academic year in both universities (April 2023 in Japan and December 2022 in Vietnam). Despite the discrepancy in the research timeline, the research methodology utilized in both countries was consistent, with the research teams undergoing prior training to minimize disparities in research approaches. There were 396 Japanese students and 2,362 Vietnamese students participating in the health checkup.

First-year students were eligible as participants because they had limited prior knowledge of cancers, making them less likely to be influenced in their responses due to their medical background. Following high school graduation, students in Japan and Vietnam can directly enroll in medical universities without holding a baccalaureate degree. As a result, the freshmen in our study had no prior medical background. Additionally, medical knowledge, including cancer-related knowledge, is not included in the university entrance exams in Japan and Vietnam. Therefore, the cancer knowledge of new-entry health science students is unaffected by these assessments.

### Survey questionnaire

The Health Service Center of Hiroshima University and the University of Medicine and Pharmacy at Ho Chi Minh City collaborated to develop the survey questionnaire. The survey took approximately 10 min to complete. The questionnaire comprised eleven questions to investigate participants’ cancer knowledge based on the guidelines of the International Agency for Research on Cancer, an affiliate of the WHO [11]. Information including the participant’s age, sex, and the health science program in which they were enrolled was also recorded. To ensure the questionnaire’s validity, the content validity index (CVI) was employed to assess the relevance and comprehensiveness of each question. The CVI score for each question was determined by dividing the number of experts who rated the question as relevant by the total number of experts. The questionnaire demonstrated good content validity, with an average CVI score of 0.79. To assess the questionnaire’s reliability, it was piloted on 15 respondents in each country, and Cronbach’s alpha coefficient was calculated. The overall questionnaire showed good internal consistency, with a Cronbach’s alpha coefficient of 0.87. The questionnaire was initially developed in English and then translated into Japanese and Vietnamese, followed by a backward translation. The original English survey questionnaire is detailed in Additional File 1.

### Ethical approval

Ethical approval for this study was obtained from the Ethical Committee of Hiroshima University, Japan (ethical number: E-143-3) and the Board of Ethics in Biomedical Research of the University of Medicine and Pharmacy at Ho Chi Minh City, Vietnam (ethical number: 822/DHYD-HDDD). Participants received a full explanation of the purpose of the study. They were assured of the anonymity and confidentiality of their participation. Informed consent was obtained from all participants. Participation in the study was voluntary, and no compensation was offered. All collected data were kept confidential and only used for research purposes. All methods were performed in accordance with the ethical standards of the institutional and national research committee and with the 1964 Helsinki Declaration and later versions.

### Statistical analysis

All statistical analyses were performed using SPSS software version 20.0 (SPSS Inc., Chicago, IL, U.S.A.). Continuous variables are summarized as the means with standard deviations (SDs), whereas categorical variables are summarized as proportions or percentages. In our study, the chi-square test of independence was applied to determine whether there was an association between categorical variables. A p-value of less than 0.05 was considered statistically significant.

## Results

### Characteristics of participants

A total of 2,748 new-entry health science students were included in this study, consisting of 394 (14.3%) Japanese students and 2,354 (85.7%) Vietnamese students. The response rates for questionnaires among the Japanese and Vietnamese students were 99.5% and 99.7%, respectively. Table 1 summarizes the characteristics of the participants. The median age of the participants was 19.1 ± 0.8 years, ranging from 18 to 31 years. The male-to-female ratio was 1:1.5. The majority of both the Japanese and Vietnamese students majored in medicine and nursing.

**Table 1:** Characteristics of the study participants

	Japanese students	Vietnamese students	Total
	(n = 394)	(n = 2354)	(n = 2748)
Age (mean ± SD)	18.5 ± 1.1	19.2 ± 0.7	19.1 ± 0.8
Male (n, %)	161 (41.5)	926 (39.3)	1087 (39.6)
Type of majors			
Medicine and nursing	239 (60.8)	1190 (50.5)	1429 (52)
Dentistry	90 (22.9)	162 (6.9)	252 (9.2)
Pharmacy	64 (16.3)	583 (24.8)	647 (23.5)
Public health	0	205 (8.7)	205 (7.5)
Chinese medicine	0	214 (9.1)	214 (7.8)

SD: standard deviations

### Knowledge about common cancers

The majority of the Japanese and Vietnamese students identified lung cancer as the most prevalent cancer, with proportions of 54.2% and 38%, respectively (Table 2). Although colon cancer is the most frequent cancer in Japan, only 19.3% of Japanese students chose it as the most common cancer. In Vietnam, liver cancer is the most common cancer, but only 33% of Vietnamese students selected this option.

**Table 2:** Knowledge about common cancers among the Japanese and Vietnamese students

	Japanese students	Vietnamese students
	(n = 394)	(n = 2354)
Lung cancer	54.2%	38%
Gastric cancer	26.5%	29%
Colon cancer	19.3%	-
Liver cancer	-	33%

### Knowledge about cancer risk factors

Table 3 presents the knowledge of the Japanese and Vietnamese students regarding cancer risk factors. Both groups had the highest understanding rates for three common risk factors, including tobacco causing pharynx cancer (95.7% and 83%, respectively), physical inactivity increasing the colon cancer risk (91.6% and 81.4%, respectively), and sun exposure raising skin cancer risk (87.8 and 77%, respectively).

**Table 3:** Knowledge about cancer risk factors among the Japanese and Vietnamese students

	Japanese students	Vietnamese students	Total	p-value
	(n = 394)	(n = 2354)	(n = 2748)	
1. Tobacco smoking increases the risk of developing pharynx cancer				
**True (n, %)**	377 (95.7)	1953 (83)	2330 (84.8)	**< 0.001**
False (n, %)	17 (4.3)	401 (17)	418 (15.2)	
2. Alcohol consumption increases the risk of esophagus cancer				
**True (n, %)**	328 (83.2)	1303 (55.4)	1631 (59.4)	**< 0.001**
False (n, %)	66 (16.8)	1051 (44.6)	1117 (40.6)	
3. *Helicobacter pylori* infection increases the risk of gastric cancer				
**True (n, %)**	316 (80.2)	571 (24.3)	887 (32.3)	**< 0.001**
False (n, %)	78 (19.8)	1783 (75.7)	1861 (67.7)	
4. Physical activity increases the risk of colon cancer.				
True (n, %)	33 (8.4)	437 (18.6)	470 (17.1)	**< 0.001**
**False (n, %)**	359 (91.6)	1917 (81.4)	2276 (82.9)	
5. Obesity increases the risk of colon cancer				
**True (n, %)**	337 (85.8)	921 (39.1)	1258 (45.8)	**< 0.001**
False (n, %)	56 (14.2)	1433 (60.9)	1489 (54.2)	
6. Hepatitis C virus (HCV) infection increases the risk of liver cancer				
**True (n, %)**	314 (79.9)	1061 (45.1)	1375 (50.1)	**< 0.001**
False (n, %)	79 (20.1)	1293 (54.9)	1372 (49.9)	
7.Estrogen–progestogen menopausal therapy decreases the risk of breast cancer				
True (n, %)	202 (51.5)	1460 (62)	1662 (60.5)	**< 0.001**
**False (n, %)**	190 (48.5)	894 (38)	1084 (39.5)	
8. Solar radiation decreases the risk of skin cancer				
True (n, %)	48 (12.2)	541 (23)	589 (21.4)	**< 0.001**
**False (n, %)**	346 (87.8)	1813 (77)	2159 (78.6)	
9. Human papillomavirus (HPV) infection increases the risk of endometrium cancer.				
True (n, %)	334 (85.2)	1826 (77.6)	2160 (78.7)	**0.001**
**False (n, %)**	58 (14.8)	528 (22.4)	586 (21.3)	

The lowest knowledge rate among both groups was the misunderstanding of the link between human papillomavirus (HPV) infection and endometrial cancer. Many students confused cervical cancer with endometrial cancer. Only 14.8% of Japanese students and 22.4% of Vietnamese students correctly replied that HPV infection and endometrial cancer were not correlated. The other low understanding rate among Japanese students was about the association between estrogen–progestogen menopausal therapy and breast cancer (48.5%), while in Vietnamese students, it was the association between *Helicobacter pylori (H. pylori)* infection and gastric cancer. Moreover, less than 50% of Vietnamese students correctly recognized the risk factors for breast, colon, and liver cancers.

Most cancer knowledge levels in Japanese students were significantly higher than those in Vietnamese students, except for the recognition of HPV infection. For this understanding, 22.4% of Vietnamese students chose the correct answer, compared to 14.8% of Japanese students (p = 0.001).

There was a statistically significant association between participants’ gender and their awareness of obesity’s role in increasing colon cancer risk (p = 0.01). More male students identified this risk factor than female students (48.7 vs. 43.9%, respectively) (Table 4). In contrast, female respondents had better knowledge about breast, skin, and endometrial cancers. The understanding rates for breast cancer were 41.1% in females and 37% in males (p = 0.02). Similarly, the recognition rates for skin cancer were 80.3% in females and 76% in males (p = 0.007). In addition, 23.4% of the female students had correct knowledge about endometrial cancer, while only 18.2% of male students had correct knowledge (p = 0.001).

**Table 4:** Knowledge about cancer risk factors separated by sex

	Male	Female	Total	p value
	(n = 1090)	(n = 1658)	(n = 2748)	
1. Tobacco smoking increases the risk of developing pharynx cancer				
**True (n, %)**	933 (85.6)	1397 (84.3)	2330 (84.8)	0.34
False (n, %)	157 (14.4)	261 (15.7)	418 (15.2)	
2. Alcohol consumption increases the risk of esophagus cancer				
**True (n, %)**	634 (58.2)	997 (60.1)	1631 (59.4)	0.3
False (n, %)	456 (41.8)	661 (39.9)	1117 (40.6)	
3. *Helicobacter pylori* infection increases the risk of stomach cancer				
**True (n, %)**	372 (34.1)	515 (31.1)	887 (32.3)	0.09
False (n, %)	718 (65.9)	1143 (68.9)	1861 (67.7)	
4. Physical activity increases the risk of colon cancer.				
True (n, %)	186 (17.1)	284 (17.1)	470 (17.1)	0.97
**False (n, %)**	903 (82.9)	1373 (82.9)	2276 (82.9)	
5. Obesity increases the risk of colon cancer				
**True (n, %)**	531 (48.7)	727 (43.9)	1258 (45.8)	**0.01**
False (n, %)	559 (51.3)	930 (56.1)	1489 (54.2)	
6. Hepatitis C virus (HCV) infection increases the risk of liver cancer				
**True (n, %)**	564 (51.7)	811 (48.9)	1375 (50.1)	0.15
False (n, %)	526 (48.3)	846 (51.1)	1372 (49.9)	
7.Estrogen–progestogen menopausal therapy decreases the risk of breast cancer				
True (n, %)	687 (63)	975 (58.9)	1662 (60.5)	**0.02**
**False (n, %)**	403 (37)	681 (41.1)	1084 (39.5)	
8. Solar radiation decreases the risk of skin cancer				
True (n, %)	262 (24)	327 (19.7)	589 (21.4)	**0.007**
**False (n, %)**	828 (76)	1331 (80.3)	2159 (78.6)	
9. Human papillomavirus (HPV) infection increases the risk of endometrium cancer.				
True (n, %)	891 (81.8)	1269 (76.6)	2160 (78.7)	**0.001**
**False (n, %)**	198 (18.2)	388 (23.4)	586 (21.3)	

In our study, there were three similar majors between the Japanese and Vietnamese students, including medicine and nursing, dentistry, and pharmacy. Meanwhile, public health and Chinese medicine programs were only available in Vietnam. Therefore, we analysed the differences in cancer knowledge between two groups of Vietnamese students: the first group included students majoring in medicine and nursing, dentistry, and pharmacy, while the second group comprised students from public health and Chinese medicine programs. Our study revealed no significant difference in cancer knowledge between these two groups, except in the awareness of the connection between obesity and colon cancer (p = 0.04).

### Sources of information about cancer knowledge

**Fig. 1:**
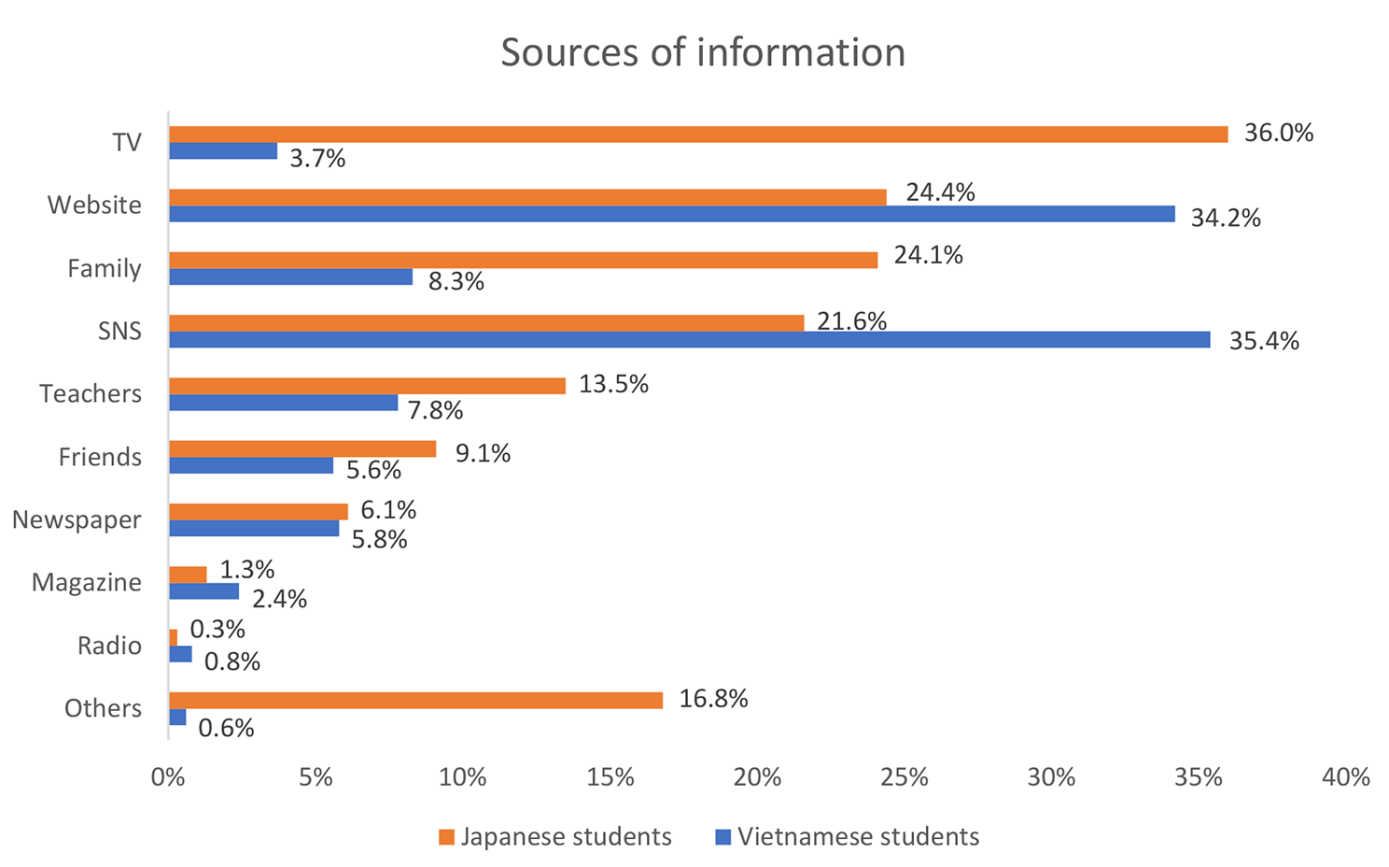
Sources of information about cancer knowledge (TV: television, SNS: Social Networking Service)

Figure 1 illustrates the sources from which the Japanese and Vietnamese students obtained cancer knowledge. Japanese students primarily gained their cancer knowledge from television (36%), websites (24.4%), and family (24.1%). Conversely, Vietnamese students predominantly relied on social networking services (35.4%), websites (34.2), and family (8.3%). In both groups, magazines, and radio were the least utilized sources of cancer information.

## Discussion

To the best of our knowledge, this may be the first questionnaire-based study investigating the knowledge of common cancers among the Japanese and Vietnamese health science students. Our study showed that the cancer knowledge levels of Japanese students were generally higher than those of Vietnamese students, except for HPV infection recognition. This difference may be because Japan has 5.6 times more new cancer cases and approximately 3.5 times more cancer deaths than Vietnam [4]. In addition, the Japanese government has made efforts to promote cancer-related education across all school grades. The Japanese Society of School Health Sciences established the Review Committee on Cancer Education in 2014 to examine cancer education-related topics from the perspective of “learning about the importance of health and life, properly managing one health, and having the right knowledge of cancer and the correct knowledge of cancer patients” [12]. Furthermore, according to the Japanese Ministry of Education, Culture, Sports, Sciences and Technology, an international survey was conducted in 2021 regarding the status of cancer education implementation nationwide [13]. This survey was conducted at 36,194 schools, including public and private elementary schools, junior high schools, compulsory education schools, high schools, secondary education schools, and special needs schools. The findings revealed that cancer education for students was carried out in 7.6% of elementary schools, 10.6% of junior high schools, and 7.1% of high schools. Meanwhile, the school education system in Vietnam places greater emphasis on infectious diseases and sex education than on cancer education [14]. This could be why Vietnamese students have less cancer knowledge than Japanese students, except for HPV infection awareness. Another reason is that Japan has implemented a population-based cancer screening program since 1982. This may also help Japanese students become more aware of cancer.

In our study, both the Japanese and Vietnamese students had limited knowledge regarding HPV infection. Previous studies also reported insufficient knowledge of HPV infection and HPV vaccines among undergraduate students, including medical students [15–20]. Furthermore, the rate of HPV vaccination coverage in Japan has experienced a significant decline since 2013, coinciding with negative campaigns against vaccination. Cervical cancer prevention was substituted with anti-vaccine content, such as information on vaccine side effects, alleged victims, and related litigation. In contrast, pro-vaccination content, such as WHO safety statements, received little media attention [21]. Therefore, the dissemination of information regarding HPV has not been observed across multiple media platforms. This may be related to the limited HPV knowledge of Japanese students in our study. As future healthcare providers, it is crucial for health science students to be well educated about HPV infection, HPV vaccination, and effective recommendations. Increasing awareness of the association between HPV and HPV-associated cancers may lead to higher HPV vaccine uptake rates. This is particularly important in low-resource countries such as Vietnam, where systematic screening programs for cervical cancer are lacking. A study examining the knowledge of cervical cancer and HPV vaccines among childbearing-aged women in Vietnam revealed an alarming lack of understanding among Vietnamese women [22]. Therefore, our findings have substantial implications for education curriculum efforts to improve HPV-related knowledge among health science students. Future campaigns in Japan and Vietnam should focus on educating students about the increasing incidence of HPV infection and cervical cancer while also emphasizing HPV vaccination as a safe and effective primary prevention strategy against this cancer.

Less than 50% of the Vietnamese students in our study were aware of the risk factors for gastric, breast, colon, and liver cancers. Remarkably, only 24.3% of Vietnamese students correctly recognized the link between *H. pylori* and gastric cancer. Vietnam is one of the Southeast Asian nations with the highest prevalence of *H. pylori* [23]. Therefore, the medical education curriculum in Vietnam should place greater emphasis on gastric cancer. In addition, universities should organize cancer-related seminars and interactive workshops for students. Currently, the Health Service Center at the University of Medicine and Pharmacy at Ho Chi Minh City has already created a website dedicated to health education topics, including cancer, for students. We hope that these initiatives will contribute to raising cancer awareness among Vietnamese students. Our study also indicated that only 48.5% of Japanese students had the correct understanding of the relationship between estrogen–progestogen menopausal therapy and breast cancer. Hence, in addition to increasing knowledge about HPV infection, medical education programs in Japan should also focus on breast cancer knowledge for students.

Our study found that female students were more knowledgeable about breast, skin, and endometrial cancers than male students. This is expected since breast cancer and endometrial cancer are female-specific cancers. Some studies also showed that female students had higher recognition of HPV infection, uterine cancer, and breast cancer than male students [10, 18, 24]. Similarly, female medical students were found to have significantly better knowledge of HPV infection than male medical students [25]. A study by Castilho et al. also demonstrated that female university students had more knowledge about the link between sunlight and skin cancer and had higher rates of daily sunscreen use than male students [26].

The sources of information about cancer knowledge also play an essential role in enhancing students’ awareness. In our study, Japanese students primarily obtained cancer information from television and websites, while Vietnamese students relied mainly on social networking services and websites. Other sources of information, such as friends, teachers, newspapers, and magazines, had lower utilization rates in both countries. These findings are consistent with another study conducted in the United States, which found that young adults predominantly sought cancer information through social media platforms [24]. Understanding these preferences and identifying reliable and user-friendly information sources is essential for effectively reaching out to young adults.

Our study has several limitations. First, the participants were recruited from only one public university for each country, and therefore, the findings may not be generalizable to the entire population of new-entry health science students. Second, there were differences in the types of majors in the Japanese and Vietnamese students. To assess the impact of these differences, we compared two groups of Vietnamese students: those with the same majors as Japanese students and those with majors exclusive to Vietnam. Our study indicated no statistically significant difference between the two groups except for the knowledge of the correlation between obesity and colon cancer. This demonstrated that the difference in majors between the Japanese and Vietnamese students had little effect on the research findings. Third, the questionnaire used in our study was relatively simple, focusing primarily on the knowledge and risk factors for common cancers. Since this study is only the first step in examining the background cancer-related knowledge of new-entry health science students, further studies should be conducted to investigate the knowledge levels of medical students as well as of young people and adults. Moreover, we expect that the study may result in reevaluating the medical education curriculum designed for health science students based on scientific evidence. Consequently, the standard institutions will review these modifications to develop more effective and appropriate medical education programs for students in Japan and Vietnam.

## Conclusion

In conclusion, the cancer knowledge levels of Japanese students were generally higher than those of Vietnamese students, except for HPV infection recognition. Both the Japanese and Vietnamese students had limited knowledge regarding HPV infection and the relationship between estrogen–progestogen menopausal therapy and breast cancer. Our study may provide valuable insights that can inform the development of medical education curricula to address inadequate cancer knowledge among Japanese and Vietnamese students.

## Data Availability

The datasets generated during and analysed during the current study are not publicly available because we did not have consent to open the data publicly from the participants but are available from the corresponding author on reasonable request.

## Abbreviations

HPV: Human papillomavirus
WHO: World Health Organization
SD: standard deviation
*H. pylori*: *Helicobacter pylori*
